# Food insecurity, social and behaviour change and distribution model: key considerations for implementation of small quantity lipid-based nutrient supplement programmes

**DOI:** 10.1017/S136898002500045X

**Published:** 2025-05-14

**Authors:** Akriti Singh, Kali Erickson, Kavita Sethuraman

**Affiliations:** 1 Helen Keller International US, New York, NY, USA; 2 USAID Advancing Nutrition, Arlington, VA, USA; 3 Independent Consultant, Guatemala, Guatemala; 4 National Cooperative Business Association CLUSA International US, Washington, DC, USA

**Keywords:** SQ-LNS, Lipid-based nutrient supplements, Child, Pregnant, Lactating women

## Abstract

**Objective:**

The objectives of this study were to (1) document factors that promoted or hindered the successful implementation of small quantity lipid-based nutrient supplements (SQ-LNS) for children 6–23 months and pregnant and lactating women (PLW) and (2) gather programme staff perspectives on considerations for expanding SQ-LNS programmes in their context.

**Design:**

We used qualitative methods to interview programme staff (*n* 23), conduct distribution site observations (*n* 9) and facilitate focus group discussions with caregivers of children 6–23 months (*n* 9) and PLW (*n* 6) with 6–8 participants per group across the three countries.

**Setting:**

The study was conducted in SQ-LNS programme sites in Honduras, Niger and Somalia.

**Results:**

We found high acceptability of SQ-LNS among caregivers of children 6–23 months and PLW women. However, caregivers and PLW were dissatisfied with the size of the product in Niger and Somalia and PLW disliked the aftertaste of iron in Honduras. In Somalia, PLW referred to high levels of food insecurity. We also found variation in how the partners designed their SQ-LNS programmes (e.g. enrolment and exit criteria), the level of communication around SQ-LNS and problem-solving to support appropriate use of SQ-LNS. Partners tracked anthropometric measurements in all countries and used the information to assess changes and, in some cases, noted improvements in child anthropometry and vaccination rates.

**Conclusions:**

Programmes need to consider several operational factors during implementation, such as securing household food access in highly food-insecure areas, counselling on the use of SQ-LNS and evidence-based criteria for enrolment, exit and supplementation duration.

Among children under 5 globally, an estimated 148 million are stunted, 45 million are wasted and 219 million do not reach their developmental potential due to undernutrition^(1,2)^. The first 2 years of life is a critical period for a child’s growth and development^(3)^. While researchers and practitioners agree that addressing undernutrition during this period requires a combination of interventions, ensuring high-quality diets during the complementary feeding period of 6–23 months is key, as is ensuring that women receive adequate nutrients during pregnancy and lactation^(4,5)^. Evidence shows that small quantity lipid-based nutrient supplements (SQ-LNS) enhance the diets of children 6–23 months and pregnant women and contribute to improved nutritional status during these life stages^(6,7)^.

SQ-LNS are food-based supplements intended to prevent undernutrition by filling nutrient gaps for children 6–23 months and pregnant and lactating women (PLW) living in areas where a nutritious diet is difficult to achieve. SQ-LNS provides energy (∼110 kcal/day), protein, multiple micronutrients and essential fats^(8)^. Data from fourteen trials show that using SQ-LNS preventively for children reduces the risk of mortality by 27 %; stunting by 12 %; wasting by 14 %; severe wasting by 31 %; iron-deficiency anaemia by 64 % and poor developmental outcomes by 16–19 %^(6,9,10)^.

Evidence on the effectiveness of SQ-LNS for pregnant women is small, but more studies are underway. A systematic review found two trials where children born to women who consumed SQ-LNS during pregnancy were longer and heavier at birth than children born to women who took iron and folic acid during pregnancy^(7)^. The same review also found that multiple micronutrient supplements and SQ-LNS had similar effects on birth outcomes, but that multiple micronutrient supplements and iron and folic acid (IFA) were more effective at reducing maternal anaemia than SQ-LNS^(7)^. It is important to note that in the studies, the amount of iron in SQ-LNS was lower than the amount of iron in multiple micronutrient supplements and IFA. Since then, a four-country randomised controlled trial showed that infants born to women who consumed SQ-LNS preconception or in the first trimester (with additional LNS if they did not meet gestational weight gain guidelines) were heavier and had lower risk of small for gestational age compared with children born to women who did not take any nutritional supplements^(11)^.

To date, SQ-LNS for children and PLW have not been part of routine nutrition programming in either development or emergency settings, thus evidence on implementation is limited^(12)^. Every year, USAID’s International Food Relief Partnership (IFRP) funds nongovernmental organisations (NGO) to programme SQ-LNS for children and PLW in emergency settings. Established in 2000, IFRP aims to provide Title II-funded specialised (ready-to-use), shelf-stable prepackaged foods, such as SQ-LNS, to improve the nutritional status of vulnerable populations^(13,14)^. Given that IFRP-funded programmes are one of the few in the world to distribute SQ-LNS, these programmes present a unique learning opportunity. In 2021 and 2022, IFRP awards provided SQ-LNS and up to USD 200 000 to transport and distribute SQ-LNS with the ability to reach up to ∼19 200 children and ∼9400 PLW over a period of 18 months^(15)^.

The 2021 *Lancet Series on Maternal and Child Undernutrition* added SQ-LNS for children to its list of proven interventions to address child undernutrition^(16)^. However, the same series also noted that currently there is no global guidance on how to optimally distribute this product within programmes. Given the lack of implementation guidance for distributing SQ-LNS in programmes, the objectives of this study were to (1) document factors that promoted or hindered successful implementation of SQ-LNS programmes based on input from programme staff and participants and (2) gather programme staff perspectives on considerations for scaling up SQ-LNS programmes in their context, including potential opportunities and constraints.

## Methods

### Study design

We used a qualitative study design and conducted key informant interviews, observations and focus group discussions (FGD) with three IFRP-funded programmes, one each in Honduras (managed by Americares), Niger (managed by Alliance for Medical Action) and Somalia (managed by GlobalMedic). Based on USAID recommendations, we selected the programmes to represent diversity in the geographic location and complementary activities (provision of food ration, routine health services *v*. comprehensive package). We collected data in Niger and Somalia in fall 2021 and in Honduras in spring 2022. More details on the findings presented in this article are available in a previously published report^(17)^.

### Study setting

In each country, an international NGO received funding from IFRP and partnered with national and local NGO to implement the SQ-LNS programme. The differences among the three programmes’ context of food security and nutritional status were not a sampling criterion, but were notable, and therefore, we describe them as background. In Honduras, the NGO provided SQ-LNS to children and PLW in five departments of the Dry Corridor: Francisco Morazán, La Paz, Valle, Comayagua and Choluteca. During the data collection period in 2022, the Famine Early Warning Systems Network (FEWS NET) predicted that departments in the Dry Corridor would experience crisis or higher levels of food insecurity (integrated phase classification [IPC] level 3)^(18)^. In 2019, the prevalence of stunting in the five departments where organisations programmed SQ-LNS was between 15 % and 35 %^(19)^. During the same year, the prevalence of underweight (BMI < 18·5 kg/m^2^) among women of reproductive age was 4 %, and the prevalence of anaemia was 22 %^(19)^.

In Niger, the NGO distributed SQ-LNS for children in Dakoro district of Maradi region. During the data collection period in 2021, the FEWS NET predicted that the Dakoro district would experience minimal levels of food insecurity (IPC level 1)^(20)^. In 2020, the prevalence of global acute malnutrition in the Maradi region was 13 %, and the prevalence of stunting was 58 % among children under 5 years of age^(21)^.

In Somalia, the NGO distributed SQ-LNS for children and PLW in the districts of Afgoye, Beletweyne, Kismayo, Mogadishu and Wanleweyn. During the data collection period in 2021, FEWS NET predicted that most of the programme implementation sites would experience crisis levels of food insecurity (IPC level 3), with one at a stressed level of food insecurity (IPC level 2)^(22)^. Al Shabab (extremist group) activity posed challenges in several programme sites (e.g. areas between Mogadishu and Kismayo)^(23)^. In 2020, the prevalence of global acute malnutrition among children under 5 years of age was 10 % to 14 %, and stunting was 27 %^(24,25)^. Additionally, 15 % of women of reproductive age were underweight, but 33 % were overweight/obese and 49 % were anaemic^(25)^.

### Data collection techniques and procedures

The lead researcher of this study interviewed programme staff from each international NGO. In each country, we hired a local consultant to conduct the interviews, site visits and FGD. Local consultants were individuals with a background in health and nutrition, who spoke the local language and were familiar with the cultural context of the study sites. The lead researcher oriented the local consultants on the study protocol, tools and research ethics focusing on asking unbiased, open-ended questions. We purposively selected programme staff, programme participants and distribution sites in consultation with the international NGO leading the programme to capture a range of experiences^(26)^. We conducted a total of twenty-three key informant interviews with programme staff representing virtually all programme managers/advisors and one staff member from each distribution site (Honduras: 8, Niger: 7, Somalia: 8) and fifteen FGD with 6–8 programme participants per group (Honduras: 6, Niger: 3, Somalia: 6). We also visited nine distribution sites (Honduras: 3, Niger: 3, Somalia: 3). In Honduras and Somalia, since the programme also distributed SQ-LNS to PLW, we divided the number of FGD among the two groups: caregivers of children 6–23 months (*n* 3) and PLW (*n* 3).

#### Interviews

During the key informant interviews (see online supplementary material, Supplementary Materials for guide), we asked programme staff, such as technical advisors, programme managers and programme officers, about implementation experiences and considerations for scale-up. Questions around implementation experiences covered how they measure success and what factors they perceived to contribute to the success of their programme as well as implementation challenges they faced. Questions around scale-up covered factors that they believe would constrain future expansion and challenges they envision with the expansion. The interviews took approximately 1 hour and were conducted virtually or in person at the office.

#### Distribution site visit

During the distribution site visit (see online supplementary material, Supplementary Materials for distribution site guide), we observed and asked programme staff at the site what was working well with the distribution and related services, what could be improved, what feedback staff received from participants and what resources would be required to expand operations. Site observations lasted several hours on 1 day and were documented. The FGD took approximately 30 min to 1 hour and were conducted in a quiet area (e.g. separate room, outside away from other people) at the distribution site.

#### Focus group discussions

Issues around how an SQ-LNS programme has been implemented can also be confirmed in terms of how participants use the product. For this reason, we conducted separate FGD (see online supplementary material, Supplementary Materials for FGD guide) with caregivers for children 6–23 months, and PLW, for the two partners who distributed SQ-LNS to PLW. The FGD were conducted on the same day as the distribution site visits. At these sites, programme staff assisted with recruiting caregivers or PLW for the FGD, from among those who were present. During the FGD, we asked how they used the product (storage, consumption, sharing, disposal of empty sachets); their motivations for giving SQ-LNS to their child or eating the product themselves; what they liked about the product and what they would change about it.

### Data analysis

The local consultants translated and transcribed the interview and FGD audio recordings into English from the language in which the consultants conducted them (e.g. French, Somali and Spanish). For analysis, the lead researcher and a second senior researcher used applied thematic analysis^(27)^. They developed a codebook, based on the themes of interest and coded the transcripts using the same codebook across the three countries, as relevant. They also coded based on emerging themes. Then, they used structured templates in Microsoft Excel to organise their findings, individually for each partner and then compared across partners. The main themes were implementation, product use and scale-up (see online supplementary material, Supplementary Materials for sub-themes).

## Results

### Programme staff description of small quantity lipid-based nutrient supplements programme

In all three countries, programme staff reported that SQ-LNS was added to an existing programme (Table 1), which is aligned with the IFRP model that intends commodities to be a complement to other existing programming. In Honduras, the existing programme for child SQ-LNS was the Ministry of Health’s Atención Integral a la Niñez en la Comunidad (Comprehensive Care for Children in the Community [AIN-C]) programme or a programme modelled after AIN-C^(28)^. The programme trained volunteers in core components of AIN-C: counselling, child growth monitoring, monitoring of pregnant women and conducting home visits. They shared that when funding was available, they also gave households food, such as MannaPack Rice^(29)^. They described measuring the weight and length of children at enrolment and exit and mid-upper arm circumference every 3 months because they did not have sufficient funds to take these measurements more frequently. For PLW, programme staff mentioned providing SQ-LNS while also monitoring pregnant women through AIN-C, health facilities and pregnancy groups. Pregnant women received food from the health facility, but staff noted that facilities were not providing the foods at the time of data collection. The programme in Honduras did not take any anthropometric measurements of pregnant women, mainly because they did not have the resources to do so, but noted that health facilities took anthropometric measurements of pregnant women.

**Table 1.** Elements of the SQ-LNS programme as described by the implementing partner staff in Honduras, Niger and Somalia

Methods	Honduras	Niger	Somalia
Implementing partners	International NGO: Americares National NGO: Honduran Order of Malta Local NGO: Hope for a Healthier Humanity, Fundación Agrolibano, Acción Honduras	International NGO: The Alliance for International Medical Action (ALIMA) National/local NGO: Bien Ètre de la Femme et de l’Enfant au Niger (BEFEN)	International NGO: The David McAntony Gibson Foundation (GlobalMedic) with a national and several local NGO
Delivery platform^*^	School, community centre, volunteer home, health facility, participant home	Health facility	Health facility
Type of frontline workers	Paid local partner staff and paid volunteers	Paid local partner staff and paid volunteers	Paid staff and unpaid volunteers
Staff to participant ratio	1:5 to 1:7	1:13 to 1:18	1:13 to 1:25
Enrolment	● Child: 6–23 months ● PLW: pregnancy	● Child: 6–11 months	● Child: 6–23 months ● PLW: pregnancy
Duration	● Child: 12 months ● PLW: 12 months	● Child: 18 months	● Child: 6 months ● PLW: 12 months
Exit	● Child: after 12 months of supplementation or after child reached 24 months of age ● PLW: after 12 months of supplementation	● Child: after child reached 24 months of age	● Child: after 6 months of supplementation ● PLW: after 12 months of supplementation
Assessment	● Child: Weight and height at enrolment and exit and MUAC every visit ● PLW: none	● Child: Height, weight and MUAC every 4 weeks	● Child: Height, weight and MUAC every 4 weeks ● PLW: weight every 4 weeks
Distribution frequency	● Every 3 months	● Every 4 weeks	● Every 2 weeks
Complementary activities	● Child: AIN-C, food ration, early child stimulation ● PLW: IFA/multiple micronutrient supplement, ANC	● Child: 1000 d programme, management of severe acute malnutrition, family MUAC	● Child: health services ● PLW: ANC, IFA

SQ-LNS, small quantity lipid-based nutrient supplement; NGO, nongovernmental organisation; PLW, pregnant and lactating women; MUAC, mid-upper arm circumference; ANC, antenatal care.

* The partner in Honduras used the community centre, school, health centre or volunteer homes as a common location to deliver SQ-LNS. Volunteers even took SQ-LNS to participants at their homes, if participants were unable to come to the distribution site.

In Somalia, programme staff said that the existing programme was primary health care services offered at the health facility. They sometimes provided SQ-LNS with other products, such as family emergency kits if their organisation had funds to programme them. They also shared challenges of providing routine health services such as vaccination, which depended on the availability of funds. Regarding anthropometric measurements, programme staff described measuring weight, length and mid-upper arm circumference of children every 4 weeks. The programme measured the weight of pregnant women every four weeks. For PLW, the programme also provided antenatal care and delivery based on funding availability. Certain health facilities in Somalia, but not the ones that distributed SQ-LNS, provided food and other products to pregnant women.

In Niger, the programme staff said that SQ-LNS was part of the organisation’s 1000 d programme, which includes antenatal care, birth delivery, neonatal care, vaccination and feeding and care from 6 months until the child reached 24 months of age^(30)^. However, they noted that these components of the programme were dependent on the availability of funds from non-IFRP sources. Programme staff did not describe giving children or their families any other food in Niger. They said that as part of the programme, they measured weight, length and mid-upper arm circumference of children every 4 weeks.

There were similarities and differences in the SQ-LNS component across the three programmes, but IFRP did not mandate any of the programme features. Programme staff shared that the SQ-LNS distribution site was a health facility in Niger and Somalia and multiple locations such as health facilities, community homes, schools, volunteer homes and even participant homes in Honduras. Staff mentioned that the programme in Niger enrolled children aged 6–11 months, whereas the programme in Honduras and Somalia enrolled any child 6–23 months. The supplementation duration for children was up to 6 months in Somalia, up to 12 months in Honduras and up to 18 months in Niger. In all three countries, programme staff reported using mid-upper arm circumference to ensure children were not suffering from wasting prior to enrolling in the programme. In Niger and Somalia, children identified as having wasting were referred to a wasting treatment programme, either at the same facility or another facility managing treatment of wasting. The programme in Honduras and Somalia enrolled pregnant women at any stage of pregnancy, and PLW received the product for up to 12 months. The programmes did not require a specific anthropometric status of pregnant women as eligibility for enrolment. They distributed SQ-LNS every 2 weeks in Somalia, every 4 weeks in Niger and every 3 months in Honduras.

### *Observation of* small quantity lipid-based nutrient supplements *distribution sites*

At the distribution sites, there was variation in the staff-to-participant ratio across the three programmes. There was a high staff to participant ratio in Honduras (1:5 to 1:7) compared with Niger (1:13 to 1:18) and Somalia (1:13 to 1:25). In Honduras, consultants observed volunteers delivering SQ-LNS related information such as the benefits and the instruction to take one sachet per day. At one site, programme staff also provided early childhood development stimulation activities. In Niger, programme staff provided nutrition education and took anthropometric measurements for the first 2 h then distributed SQ-LNS. In Somalia, our consultant did not observe the standard provision of SQ-LNS-related information, and caregivers typically chatted among themselves as they waited for their SQ-LNS ration. The programme staff in Niger and Somalia counted the number of SQ-LNS sachets before handing them over to each participant at the distribution site whereas programme staff in Honduras handed over bags of individual rations, which they had prepared in advance. Consultants observed participants returning empty sachets at only one site each in Niger and Somalia and none in Honduras. Staff in Honduras collected the sachets in advance of distribution day whenever they met them either at home or elsewhere.

### *Programme staff perceived strengths of* small quantity lipid-based nutrient supplements *programme*

Across the three countries, programme staff described several strengths of their SQ-LNS programme (Table 2). They perceived high acceptability of SQ-LNS among programme participants. They also mentioned observing positive changes after adding SQ-LNS to their programme, such as fewer underweight children in Honduras and higher vaccination rates and lower cases of severe acute malnutrition in Niger based on project data. The programme in Somalia had just begun implementation when we collected data, thus they could not speak to any observed changes as a result of the SQ-LNS programme.

**Table 2.** Programme staff perceived strengths and challenges

	Honduras	Niger	Somalia
Perceived strengths			
Results	Programme resulted in fewer underweight children	Programme resulted in higher vaccination rates, lower cases of severe acute malnutrition	(Programme had just begun implementation during data collection for this study)
Illustrative quotes	“Yes, [SQ-LNS] supported us a lot, the cases [of underweight children] have been reduced greatly.” — *Local NGO staff*	“It is measured over time; it has raised the indicators of routine immunisation and reduced the admissions of children 6–23 months into the severe acute malnutrition [treatment] program” — *Local NGO staff*	Not applicable (N/A)
Successes	● Capable local NGO ● Implementation through a large network of volunteers was working well ● Applied lessons learned from prior experience implementing programme in Guatemala	● Applied lessons learned from prior experience implementing programme in Mirriah District	● High staff capacity ● Programme participants using SQ-LNS correctly
Illustrative quotes	“We shared the information and the experiences of lessons learned in Guatemala with Honduras.” — *Local NGO staff*	“So, we had lessons learned [from Mirriah], which allowed us in any case not to repeat the same mistakes in Dakoro.” — *Local NGO staff*	“I would say this was contributed by our staff who do regular follow up with the beneficiaries, also health promotion messages played an important role; additionally, the returning of the empty sachets was also a crucial part of the success as this has prevented mothers from selling the product in the market as other products.” — *Local NGO staff*
Supervision	Department coordinator/promoters closely monitor volunteers	Not mentioned	Close supervision from programme focal person every month, monitoring and evaluation (M&E) officer every quarter
Illustrative quotes	“The departmental coordinator is almost daily with them solving problems.” — *International NGO staff*	N/A	“Ongoing monitoring visits by the [local NGO] project focal person (I visit every distribution site once a month, and our M&E officer visits the distribution sites on a quarterly basis).” — *Local NGO staff*
Supply chain	Experience and system in place at the national level to move large volumes of different types of products	Strong system in place (importation, warehouse, transport to health centers and health posts)	Not mentioned
Illustrative quotes	“[Local NGO] has a very, very big warehouse. They are able to manage around 25, 40-foot containers at once.” — *International NGO staff*	“Logistics are fine! So, as we said, the product comes from the United States. Our logistics supply chain is ensured and the products are transited through Benin.” — *Local NGO staff*	N/A
Participants	High acceptability of SQ-LNS	High acceptability of SQ-LNS	High acceptability of SQ-LNS
Illustrative quotes	“[A] lady told us, ‘I had a baby before and I had problems because I did not get milk with the previous pregnancies, and now since I have taken [SQ-LNS], the child came out with good weight, beautiful skin and healthy.’ Besides that, when they put him to breastfeed, she says, ‘I got a lot of milk. In my life I have never had as much milk as now.’” — *Local NGO staff*	“I would ask [caregivers] what they thought about SQ-LNS and they would just hold up their kid and [say]…look at his hair, look at his skin, just look at him…” — *International NGO staff*	“The beneficiaries are enthusiastic about the product and frequently share with us how happy they are; the product is also widely accepted, and they enjoy it so much that they are asking for more.” — *Local NGO staff*
Perceived challenges			
Funding amount	● Insufficient to hire required number of staff ● Insufficient to print social and behaviour change materials	● Insufficient to hire required number of staff	● Insufficient to hire required number of staff ● Insufficient to print communication materials
Illustrative quotes	“Yes, because this is the limiting factor, there is no budget to make flipcharts for everyone, we only have one.” — *Local NGO staff*	“So, if we had more staff, maybe moms won’t come in the morning and leave in the afternoon. They will be served very quickly and go about their daily duties.” — *Local NGO staff*	“We don’t have a budget [for] IEC [information, education, and communication] materials, additionally we don’t support a good number of staff to provide other complementary activities.” — *Local NGO staff*
Supply chain	● Delay in arrival of product from United States ● Higher than expected transportation cost (fuel price)	Not mentioned	● Delay in arrival of product from United States ● Transportation to areas affected by Al Shabab activity
Illustrative quotes	“The first problem that we faced was delay on the product being in Honduras, we signed an agreement with USAID about six or nine months before the commodities really got to Honduras.” — *International NGO staff*	N/A	“It’s difficult to transport products from warehouses to distribution centres because there are so many security checkpoints throughout the city, and security personnel won’t let you pass without checking your ID card, which can take a long time, and sometimes there will be a complete city shutdown with no traffic.” — *Local NGO staff*
Storage	Not mentioned	Storage of SQ-LNS at health centres and health posts because the product was not recognised by the Nigerien government	Not mentioned
Illustrative quotes	N/A	“The challenge was the place of storage because the national supervision decided not to put the [SQ-LNS] and the other nutritional inputs in the same store because it is not recognized by the Nigerien state.” — *Local NGO staff*	N/A
M&E	Delay in data entry	Not mentioned	Delay in data entry
Illustrative quotes	“They will have to take one or two weeks before they can come to the office and then give the papers to someone there who will then collect all these papers and upload the information to the system.” *— International NGO staff*	N/A	“Also, the staff is insufficient. Two staff can’t cover the workload, such as screening, identification, registration, distribution, and data entry to the Excel sheet.” *— Local NGO staff*
Complementary activities	Could not take anthropometric measurements more frequently (only measured length and weight at enrolment and exit, and MUAC every 3 months)	Dependent on non-IFRP funding	Dependent on non-IFRP funding
Illustrative quotes	“It is very difficult also to measure every child, take the anthropometric measurements of every child every three months, because there are just so many children registered into the project.” — *International NGO staff*	“The challenges were more related to the budget envelope which was insufficient, which will not allow us to cover the 18 months as planned in the project.” —*Local NGO staff*	“The service that we would like to provide depends on the funds we receive from donors.” — *Local NGO staff*
Participants	● Caregivers and PLW dislike the aftertaste of iron	● Caregivers visiting multiple distribution sites ● Caregivers found the size too small	● Caregivers and PLW found the size too small
Illustrative quotes	“Children are eating [SQ-LNS] as it is like peanut butter, they feel it is tastier. The mothers say no, that it’s a little bitter, that’s how they feel.” — *Local NGO staff*	“They say that we only thought of the children and that the size of the sachet is very small. If only we could bring big sachets so that the mothers could enjoy them as well as the other children.” — *Local NGO staff*	“When we started the project, the beneficiaries asked us questions about the size of [SQ-LNS]. After discussing with them, now they are accepting the quality of the product over the size.” — *Local NGO staff*

Other perceived strengths included high staff capacity and learning from their prior implementation experiences, allowing them to make necessary modifications to future SQ-LNS programmes to ensure they ran smoothly. Programme staff in Honduras and Somalia also reported that they had a strong supervision plan, and programme staff in Honduras and Niger noted that they had a strong logistics system in place to move large volumes of SQ-LNS across distribution sites.

In Honduras, several volunteers described problem solving with PLW. They shared that if a pregnant woman says she does not feel like eating SQ-LNS, they encourage her to eat small portions over the course of the day, or eat SQ-LNS with food, milk or water. They also mentioned telling pregnant women about the benefits of SQ-LNS: the product will provide strength to deliver the baby and that the baby would be born beautiful. For those who forget to take SQ-LNS, they described making reminder Post-It notes or designating someone to ensure that the woman took her supplement. Volunteers in Honduras described conducting home visits.

### Programme staff perceived challenges of their small quantity lipid-based nutrient supplements programme

Programme staff also encountered implementation challenges (Table 2). Staff in Niger and Somalia mentioned that the most common issue was that caregivers of children 6–23 months and PLW found the size of SQ-LNS too small. Staff in Honduras mentioned that while PLW did not comment on the size of the product, both PLW and caregivers mentioned a strong dislike for the aftertaste of iron after consuming SQ-LNS. Despite this, PLW reported consuming SQ-LNS, and caregivers reported giving SQ-LNS to their children.

Another common challenge was how to better communicate information about SQ-LNS with programme participants. In Honduras, one of the local partner NGO had developed a SQ-LNS flipchart for volunteers to use with programme participants. However, the local NGO did not have the funds to print enough copies for all 150 volunteers. Similarly, programme staff in Somalia mentioned that they would like to print communication materials for programme staff to provide accurate information to programme participants about SQ-LNS but did not have funds to do so; they shared some information on how to use SQ-LNS verbally. The implementing partner in Niger agreed that communication materials would be helpful.

In Somalia, programme staff noted that participants who lived further away from the distribution site requested monthly distribution, as opposed to the current 2 weeks. In Honduras, programme staff suggested distribution every 4 months in very remote areas, as opposed to the current 3 months, due to poor road conditions making it challenging to transport SQ-LNS more frequently.

Other challenges implementing partners shared about adding SQ-LNS to their existing programme included insufficient funding to hire the required number of staff for programming in all three countries, and for data entry in Honduras and Somalia; and difficulty ensuring uninterrupted transport of SQ-LNS in certain areas due to extremist group activity in Somalia. The manufacturer in the US ships SQ-LNS to the countries where the programmes operate. However, programme staff in Honduras and Somalia mentioned implementation setbacks due to COVID-19 pandemic-related SQ-LNS production and transport delays. The Niger programme had left over SQ-LNS from the previous year so did not experience the same issues.

### Experiences of caregivers of children 6–23 months who received small quantity lipid-based nutrient supplements

In Honduras, Niger and Somalia, caregivers of children 6–23 months described an overwhelmingly positive response towards SQ-LNS (Table 3). They noted that their children mostly ate SQ-LNS as is, while a few caregivers in Somalia mentioned that they mixed the product with milk or water for their children. Caregivers primarily shared that they gave only one sachet of SQ-LNS per day, as recommended, to their children, while a few caregivers in Somalia mentioned that they gave their children more than one sachet because their children asked for more. All caregivers in Honduras mentioned that they returned empty sachets of SQ-LNS to the distribution site, while only some caregivers in Niger and Somalia mentioned that they did so. Implementing partners asked participants to return empty sachets to track compliance and to appropriately dispose of the sachets.

**Table 3.** Experiences of caregivers of children 6–23 months and pregnant and lactating women

	Honduras	Niger	Somalia
Caregivers of children 6–23 months			
Use	● Child ate as is ● Child ate 1 sachet per day ● Returned empty sachets	● Child ate as is ● Child ate 1 sachet per day ● Some returned empty sachets	● Child ate as is, some mixed with milk or water ● Most gave 1 sachet per day, but some caregivers gave 2–3 sachets per day ● Some returned empty sachets
Benefits	● Child has greater appetite ● Child has gained weight and height ● Child has increased level of activity ● Child is well-nourished	● Child is happy ● Child is healthy ● Child grows well	● Child drank more water ● Child has gained weight ● Child has more energy ● Child looks good
Illustrative quotes	“Before giving him this product, the child didn’t eat like that. He didn’t ask me so much. There, one sees how it helps them a lot, their appetite.” — *Caregiver, La Paz*	“We observe that the child who consumes [SQ-LNS] thrives better than another child who does not consume it.” — *Caregiver, Baban Kori*	“I can feel it from my child that he is doing well, and his weight and his overall energy are better and even his appetite is better now.” *— Caregiver, Mogadishu*
Feedback	● Dislike after the taste of iron ● Improve packaging ● Give SQ-LNS to children beyond 24 months ● Manage distribution sites better	● Size is small ● Enrol children older than 11 months, ● Want more information on SQ-LNS ● Manage distribution sites better	● Size is small ● Want more time with the clinic staff ● Want 1 month supply for those who live far ● Want complementary activities (ANC, delivery care, vaccines)
Illustrative quotes	“One must be aware of giving a glass of juice or milk, for the taste in the mouth. Because [after] finishing the packet, just 15 min after, one has an iron taste.” *— Caregiver, La Paz Department*	“We only want the LNS-SQ partner to increase the [LNS-SQ] size. Otherwise, the taste, color, and packaging are good.” *— Caregiver, Kornaka*	“I will say to increase the size of the product, it’s very small and it’s not sufficient even for the children.” *— Caregiver, Mogadishu*
Pregnant and lactating women			
Use	● Some mixed with food or drink ● Some ate more than one sachet per day ● Took in addition to IFA/MMS, when available	(This programme did not provide SQ-LNS to PLW)	● Ate as is ● Most ate more than one sachet per day ● Some also took IFA and corn-soy blend
Benefits	● Improved appetite ● Some said babies were born “beautiful” and “chubby” ● Others said several factors were involved in the benefits they observed to the baby	NA	● More energy, greater appetite, less constipation, slight increase in weight, drank more water
Illustrative quotes	In my case, the other girl was born underweight. This one [delivered after consuming SQ-LNS] was born with a good weight. — *PLW, La Paz Department*	NA	“Once you start eating this product, you won’t be able to stop, since it tastes so good and has so much nourishment that your body will feel energized.” — *PLW, Mogadishu*
Feedback	● Disliked after the taste of iron ● Difficult to open sachets ● Enjoy group meetings ● Provide inputs for kitchen gardens	N/A	● Size is small; also give porridge ● Want more time with staff ● Difficult to return empty sachets
Illustrative quotes	“If you only eat [SQ-LNS] and you don’t eat well, you don’t eat fruits, vegetables, vitamins, all that, you won’t benefit from it” *— PLW, Choluteca Department*	N/A	“I also eat porridge from another [maternal and child health clinic] which I am registered as pregnant because they give us enough food which I can eat, and it can fill my stomach but the LNS can’t fill my stomach.” *— PLW, Mogadishu*

The majority of caregivers described benefits from their children consuming SQ-LNS, including that their children had gained weight, were energetic and looked good. When asked for feedback about the product, caregivers in Niger and Somalia commented on the small size of SQ-LNS while caregivers in Honduras commented on the aftertaste of iron. The caregivers in Honduras did not specify if their children made this comment or they had tried the product and felt their children would also experience the same aftertaste. Despite this, they noted that their children ate the product anyway. Other points of feedback from caregivers included a request for more time with programme staff to learn about SQ-LNS, including why it differs from other LNS products in Somalia and Niger, monthly distribution for those who lived further away in Somalia (where the frequency of distribution was every 2 weeks) and complementary services such as antenatal care and vaccination in Somalia. Caregivers also requested that the distribution sites be better managed: sites should have benches for everyone in Niger and Somalia and guaranteed use of community sites during distribution day in Honduras, as they were unable to use them at times.

### Experiences of pregnant and lactating women who were taking small quantity lipid-based nutrient supplements

The programmes in Honduras and Somalia also distributed SQ-LNS for PLW (Table 3). In the two countries, most PLW described eating SQ-LNS as is, but some in Honduras mentioned that they mixed it with food or drink. While few women in Honduras shared that they ate more than one sachet of SQ-LNS per day, most PLW in Somalia described eating more than one sachet per day and even up to 5 because they enjoyed it or because no one had told them how many to eat per day.

PLW in both countries shared benefits, including an increased appetite after consuming SQ-LNS. PLW in Somalia mentioned that they drank more water. Some PLW in Honduras, where the programme had been implemented for almost a year prior to data collection for this activity, mentioned that babies born after the mother consumed SQ-LNS during pregnancy were ‘beautiful’ and ‘chubby,’ while other PLW mentioned that several factors (eating fruits and vegetables and taking prenatal vitamins) were involved in ensuring positive birth outcomes. When asked for feedback, PLW in Honduras commented on the aftertaste of iron and a few mentioned that it was difficult to open the sachets. The majority of PLW in Somalia commented on the small size of SQ-LNS, noting that it was too small even for children. They requested porridge, adding that this would ‘fill their stomach,’ which a small sachet of SQ-LNS would not. They also mentioned that it was difficult to return the empty sachets.

### *Programme staff perspectives on scale-up of* small quantity lipid-based nutrient supplements

Programme staff in all three countries believed they could scale up their SQ-LNS programme. However, they noted that more funding, adequate human resources, better storage capacity in the communities and improved monitoring and evaluation systems would be necessary to implement their SQ-LNS programme at scale. Programme staff also shared that expansion through the health system would be ideal, such as through the health facilities in Niger and Somalia and the AIN-C programme in Honduras. Programme staff in Niger mentioned that it would be critical to consider developing biodegradable SQ-LNS packaging, as it was challenging to return and dispose of empty SQ-LNS sachets.

## Discussion

In this study conducted with SQ-LNS programme staff and participants in Honduras, Niger and Somalia, we found high acceptability (e.g. reported consistent consumption and perceived benefits) of SQ-LNS among caregivers of children 6–23 months and PLW. However, caregivers and PLW were dissatisfied with the size of the product in Niger and Somalia and disliked the aftertaste of iron in Honduras. In Somalia, PLW referred to high levels of food insecurity. We also found variation in how the partners designed their SQ-LNS programmes, e.g. not all programmes began supplementation at close to 6 months of age, when evidence shows the product is most effective. The level of communication around SQ-LNS and problem solving to support appropriate use of SQ-LNS among caregivers and PLW varied across the programmes. Further, while critical to the health and nutrition of children and PLW, provision of complementary activities such as primary health care services and provision of food assistance (in highly food insecure areas) depended on the partner’s access to non-IFRP sources of funding, which was variable across the three sites. Finally, although not required by IFRP, partners tracked anthropometric measurements and used the information to assess changes and, in cases, noted improvements in child anthropometry and vaccination rates.

Consistent with the findings of our study, previous studies in Sub-Saharan Africa have also reported high acceptability of SQ-LNS among children and PLW^(31–35)^. However, few studies have documented the acceptability of SQ-LNS in Latin America and the Caribbean region. One noted high acceptability of SQ-LNS among children in Guatemala^(36)^. Another found high compliance of SQ-LNS among pregnant women in Guatemala^(11)^. While we were unable to find a study from the Latin America and the Caribbean region that reported similar findings about the dislike for the aftertaste of iron as shared by PLW in Honduras, a study in Ghana reported that some PLW did not react positively to the smell and taste of SQ-LNS^(34)^. These Ghanaian PLW noted that they could smell and taste the ‘medicine,’ and some said a bitter aftertaste made it difficult to consume the SQ-LNS^(34)^. It is important to note that the amount of iron in the SQ-LNS for PLW is much higher than the amount of iron in the SQ-LNS for children.

Studies conducted in development contexts have contributed to the evidence on the effectiveness of SQ-LNS. A meta-analysis of these studies found that household food insecurity did not modify the impact of SQ-LNS on child growth, suggesting that SQ-LNS is effective even among children living in food-insecure households^(37)^. However, the level of food insecurity in SQ-LNS programme areas in Somalia is more serious (IPC 3, crisis levels) than the settings of the studies included in the meta-analysis. While the SQ-LNS programme sites in Honduras and Somalia were both at crisis levels of food insecurity, caregivers and PLW in Honduras did not complain about the small size of SQ-LNS, possibly because they were receiving food assistance (through a non-IFRP funding mechanism) and/or the health facility. UNICEF’s recent guidance note on programming child SQ-LNS encourages implementing partners to programme SQ-LNS in areas at IPC 3–5 levels only if they provide household food access interventions such as food or cash^(38)^. This guidance is important because SQ-LNS is not a replacement for food but is designed to enhance the quality of existing diets.

Evidence from SQ-LNS trials for children suggests that the greatest benefits are obtained when children begin consuming the product at 6 months of age and continue for at least 12 months^(39)^. We found that there was variation across the three implementing partners in terms of enrolment criteria, exit criteria and supplementation duration. The enrolment criteria for children in Niger was between 6 and 11 months while in Honduras and Somalia, the enrolment criteria sought children between 6 and 23 months. The duration of supplementation was up to 18 months in Niger, 12 months in Honduras and 6 months in Somalia. In all countries, children were no longer eligible to receive the product once they reached 24 months of age. Several of these design decisions were driven by funding constraints.

Researchers emphasise that appropriate infant and young child feeding counselling must accompany SQ-LNS programming^(6)^. However, in our study, we found that this was not always happening. Given the large volume of programme participants on distribution day, staff in Niger and Somalia were unable to share information with all participants on how to use SQ-LNS. As a result, some caregivers in Somalia had questions about why SQ-LNS differed from other LNS products used to treat wasting. However, programme volunteers in Honduras interacted with a small group of participants on a regular basis and received training on how to counsel. USAID has taken learnings from this study into account in updating the fiscal year 2024 request for proposal for IFRP awards^(40)^. While several published papers document social and behaviour change communication approaches for SQ-LNS, there is currently no global set of counselling cards or job aids for programme staff to use^(41,42)^. Forthcoming operational guidance from the SQ-LNS Task Force and sample job aids from USAID Advancing Nutrition will support implementing partners in designing their communication plan for SQ-LNS programmes^(43,44)^.

SQ-LNS is a highly effective product, but questions remain about how to scale it up. As programme staff noted in this study and demonstrated through their programmes, it is possible to expand implementation of SQ-LNS through the health system by leveraging existing platforms such as growth monitoring and promotion and antenatal care. However, they highlighted several points to consider when scaling up, such as ensuring storage capacity at the local level. The social protection system may be another approach to distribute SQ-LNS. The World Bank recently published a policy note on programming SQ-LNS through the Sahel Adaptive Social Protection Program^(45)^. It is also essential to engage with country governments to understand how they might choose to finance and implement SQ-LNS. At this time, organisations source SQ-LNS from the United States or Europe, but supporting regional/local production would be critical to strengthening the supply chain and therefore the sustainability of the programme. Adding the product to the WHO Essential Medicines List, as has been done for some LNS products, and national medicine lists, would enable country governments to procure the products through their medical commodities budgets^(46)^. This will also permit the sale of SQ-LNS, making it more accessible.

Future studies should document how to scale up high-quality SQ-LNS programmes in different settings as well as how to deliver SQ-LNS in the presence of other nutritional supplements for children and for women to prevent toxicity. Additionally, research on the impact of SQ-LNS on pregnant women has largely focused on the child, but should also focus on the impact on the mother during pregnancy (in addition to anaemia), as well as postpartum^(47)^. In this study, mothers perceived some benefits for themselves (e.g. increased energy, appetite, water consumption), but these would need to be empirically examined.

There were several limitations to this study. First, since we only conducted the study in three settings, the findings may not be generalisable to all SQ-LNS programmes because each programme’s context varies. Second, in Niger and Somalia, male consultants spoke to female participants, which could have influenced the quality of the data we received. In Honduras, where data collection occurred after the other two countries, a female consultant was hired to conduct the FGD. Third, in Honduras, programme volunteers attended the FGD with participants, and national NGO staff attended interviews with local NGO staff. We permitted these individuals to join the FGD and interviews because we designed the study as a collaborative activity. We recognise that due to this, programme participants and local NGO staff may have refrained from speaking freely. Fourth, we did not gather information on whether programme participants received food assistance from other sources, which could have influenced their response to SQ-LNS. Finally, there may have been inherent response bias since USAID funded this study and the IFRP programmes, thus programme staff and participants may have wanted to portray the study in a positive light.

Despite these limitations, this study is significant because we documented implementation successes and challenges from IFRP-funded SQ-LNS programmes, one of the few in the world that distribute SQ-LNS in a real-world programme context. Further, we explored programme experiences in three diverse contexts and found similar challenges, which highlights the need for greater clarity and guidance on SQ-LNS programme implementation. Specifically, more information is needed on how to layer and sequence SQ-LNS with other programme activities, ensure supplements are provided for an optimal duration to be beneficial and set an appropriate distribution frequency, while also strengthening the social and behaviour change aspects of the programme. These findings have and will continue to inform SQ-LNS programme implementation and global operational guidance.

## Supporting information

Singh et al. supplementary material Singh et al. supplementary material

## Supplementary material

For supplementary material accompanying this paper visit https://doi.org/10.1017/S136898002500045X
