# Supplementary material for: Food insecurity, social and behaviour change and distribution model: key considerations for implementation of small quantity lipid-based nutrient supplement programmes
Source: Public Health Nutr. 2025 May 14;28(1):e78. doi: 10.1017/S136898002500045X (PMC12100554; doi:10.1017/S136898002500045X)
Supplement: Singh et al. supplementary material [file S136898002500045Xsup001.docx]

**Supplemental Materials**

1. **Interview guide**

Informed Consent

My name is ____ and I am working on behalf of USAID Advancing Nutrition. USAID Advancing Nutrition is a global nutrition project funded by the United States Agency for International Development (USAID). We are conducting a study in ___ to learn about USAID’s International Food Relief Partnership (IFRP)-funded programs that provide small quantity lipid-based nutrient supplement (SQ-LNS) to children 6–23 months of age. The results of this study will inform the design of future LNS programs.

We would like to gather information about your experiences and perspectives on the implementation of SQ-LNS programs and considerations for expanding program operations. The discussion will take about one hour to complete. Your participation is entirely voluntary. You can decline to participate without any impact on your employment or the IFRP award that your organization currently receives or may receive in the future. You are free to not answer certain questions or to stop participating at any time without any penalty. There is no direct benefit for participating.

We will keep any personal information that you give us, such as your name, confidential and will only share it with the study team. The study team does not include USAID. We will remove your personal information before sharing the information you give us outside the study team. In study reports and presentations, we will combine the information you provide us with the information we gather from others. Approximately ___ people will participate in this study. We will share the combined information with USAID and may share it with government officials if they wish to see it. This is not an evaluation of your organization’s performance.

Do you have any questions about participating?

- If yes (Y), answer any questions.
- If no (N), move to the next question.

Do you agree to participate?

- If yes, thank them for agreeing to participate and move to the next question. For focus group discussions, ensure that each person agrees to participate.
- If no, thank them for their time and politely leave.

Can we audio record the conversation?

- If yes, proceed with audio recording. For focus group discussions, ensure that each person agrees.
- If no, say that it is no problem and proceed without audio recording.

If you have any questions about the study, you may contact [the study manager] at ____.

| ID (LNS_Country_PI_Sequence#) |  |
| --- | --- |
| Location |  |
| Respondent sex |  |
| Respondent organization |  |
| Respondent position |  |
| Length of time with IFRP LNS program |  |
| Date of interview |  |
| Interviewer name |  |
| Notetaker name |  |
| Language interview conducted in |  |
| Consent to interview (Y/N) |  |
| Consent to audio record (Y/N) |  |

**IFRP SQ-LNS program experience**

1. Please describe your IFRP SQ-LNS program.

*Probes:*

- Coordination: Which nutrition actors^^[[1]](#footnote-1)^^ do you coordinate with (national, regional, local)?
- Complementary activities: Which complementary activities^^[[2]](#footnote-2)^^ do you implement along with the IFRP SQ-LNS program at the time of distribution?
- What program is your IFRP work a complement to (larger program within which SQ-LNS program is embedded)?
- Social and behavior change (SBC) communication: What do program staff tell program participants about SQ-LNS (why to use, how to use, number to take per day, side effects etc.)?
- How do you deliver the information about the product (verbally, printed materials, group sessions, etc.)?
- Monitoring and evaluation (M&E): What does your M&E system for the IFRP SQ-LNS program look like [1) format: paper-based, electronic; 2) tools: stock cards, register, ration card etc.; 3) indicators: # reached, stunting, wasting etc.]?

— What data do you collect?

- Which anthropometric measurements, if any, do you take (length, weight, MUAC, head circumference)? Does this vary for children and pregnant and lactating women?
- Which developmental milestones, if any, do you measure (waking, speaking, interacting with others)?
- How do you use the data you collect? How frequently do you use the data collected?
- Distribution model: What is the distribution model of your SQ-LNS program (e.g., targeting, duration, frequency)?

— How many years have you been implementing the IFRP SQ-LNS program?

— Has the model changed over the years (participant eligibility criteria)?

- Human resources: How many program staff are involved in the IFRP SQ-LNS program (program manager, warehouse manager, guards, distribution agents, volunteers, M&E officer)?
- What type of training do they receive (number of days, content of training, frequency of training)?
- Do the program staff get any written information about how to share information on the use of SQ-LNS with the program participants?

2. What successes has your IFRP-LNS program had to date? What is going well?

*Probes:*

- How are you measuring success?
- What factors contributed to this success?
- What has surprised you?

3. What challenges in implementing the IFRP SQ-LNS program have you faced so far? What has been difficult?

*Probes:*

- Start-up phase: Were there any delays? How did you address them?
- Product use: How do you ensure that participants use SQ-LNS as intended?
- Product acceptability: Have the products been acceptable (taste, ease of use, ease of transportation, etc.) to the participants and national government?
- Logistics: What challenges did you encounter with importing, storing, or transporting SQ-LNS?
- Coordination with nutrition actors: What challenges in coordinating with nutrition actors have you experienced? Which nutrition actors did you try to coordinate with in the past? What happened? Which nutrition actors would you like to coordinate with now or in the future?
- Complementary services: What challenges with providing complementary services have you experienced? Which services did you try to provide? Which services would you like to provide now or in the future? Why?
- SBC communication: Have there been any challenges in communicating or sharing information about SQ-LNS? Have you had to alter what you communicate with program participants? If yes, what did you have to alter and why?
- Anthropometric measurements: What would enable you to overcome these challenges?
- Have you tried something to improve or overcome the challenges you mentioned that did not work? What was effective at helping you overcome the challenges?

4. How have you considered inclusion of children or children of caregivers with a disability in your program design and implementation?

*Probes:*

- Do children or adults with disabilities regularly participate in SQ-LNS distributions? Why or why not?
- What challenges have you experienced ensuring that children and caregivers with disabilities are included in your program?
- What could be done to promote greater inclusion of children and adults with disabilities?

**Considerations for scaling-up**

5. Are you currently utilizing the maximum amount of IFRP SQ-LNS resources available to you? What currently limits the scale of your SQ-LNS program?

*Probes:*

- Team/organizational bandwidth
- Logistics
- Infrastructure
- Funding

6. If you were to expand your current SQ-LNS program, what size would you be able to expand to?
 *Probes:*

- Geographic coverage (e.g., district), number of participants (children)
- What factors would influence this decision to expand operations (funding, staffing)?
- What would you need (funding, staffing) to be able to cover all eligible children in your working area?
- Would the current program model need to change (e.g., targeting, delivery platform, services provided)?

7. Which stakeholders would you need support from to operate at a larger scale?

*Probes:*

- Government, IFRP, other nutrition actors, community
- What type of support would you need from the stakeholders you mentioned (funding, human resources, M&E system, etc.)?

8. How might a shift in scale affect your M&E system? What might you need to do differently?

Probes:

- Which indicators would you use?
- Which indicators other than anthropometric measurement might be appropriate (child development, maternal time, maternal mental health, etc.)?
- How would your M&E data management systems need to change?

9. What potential challenges do you see for operating at this scale?

*Probes:*

- Storage, transportation, distribution, complementary activities, quality
- How would you ensure program quality when operating at this scale? (guiding documents, supervision, supply of SQ-LNS [sustainability])
- Are there opportunities for local production of SQ-LNS? By the private sector?

10. In your opinion, is SQ-LNS an appropriate product for the population that your organization serves given the level of food insecurity in the area? Why or why not?

11. Is there anything else you would like to share with us regarding your program implementation experience or considerations for expanding program operations?

**2. Distribution site guide**

Informed Consent

My name is ____ and I am working on behalf of USAID Advancing Nutrition. USAID Advancing Nutrition is a global nutrition project funded by the United States Agency for International Development (USAID). We are conducting a study in ___ to learn about USAID’s International Food Relief Partnership (IFRP)-funded programs that provide small quantity lipid-based nutrient supplement (SQ-LNS) to children 6–23 months of age. The results of this study will inform the design of future LNS programs.

Today we will document how your program distributes SQ-LNS and provides other related services. Is it okay if I observe the session today? I also have a few questions related to your work that I would like to ask you. Can I ask these questions after you have completed your tasks for the day? The discussion will take about 30 minutes. Your participation is entirely voluntary. You can decline to participate without any impact on your employment or the IFRP awards your organization currently receive or may receive in the future. You are free to not answer certain questions or to stop participating at any time without any penalty. There is no direct benefit for participating.

We will keep any personal information that you give us, such as your name, confidential and will only share it in the study team. The study team does not include USAID. We will remove your personal information before sharing the information you give us outside the study team. In study reports and presentations, we will combine the information you provide us with the information we gather from others. Approximately ___ people will participate in this study. We will share the combined information with USAID and may share it with government officials if they wish to see it. This is not an evaluation of your organization’s performance.

Do you have any questions about participating?

- If yes, answer any questions.
- If no, move to the next question.

Do you agree to participate?

- If yes, thank them for agreeing to participate and move to the next question. For focus group discussions, ensure that each person agrees to participate.
- If no, thank them for their time and politely leave.

If you have any questions about the study, you may contact [the study manager] at ____.

| ID (LNS_Country_DG_Sequence#) |  |
| --- | --- |
| Location |  |
| Start time |  |
| End time |  |
| Respondent sex |  |
| Respondent organization |  |
| Respondent position |  |
| Length of time with IFRP LNS program |  |
| Date of observation/interview |  |
| Interviewer name |  |
| Notetaker name |  |
| Language interview conducted in |  |
| Consent to observe/interview (Y/N) |  |

**Observations**

1. Type of place where you are observing the distribution of SQ-LNS?

*[Health facility, school, church, etc.]*

2. How many people are involved in the distribution/ providing related services at this site?

*Probes:*

- What roles do partner staff, government staff, community leaders, or volunteers play?
- If possible, describe the number by role.

3. How would you describe the participants that have shown up (at the beginning/when the data collection begins)?

*Probes:*

- How many people (other than children) have shown up?
- Are they generally male or female?
- In addition to parents, how common is it that other caretakers come for the distribution?
- Do caretakers usually bring young children?
- Do they show up at a designated time or throughout the day?

3a. Are there any participants with a disability, either a child or the caregiver of a child who has a disability present?

*Probes:*

- If yes, please describe the disability (if possible) and how the participants are accommodated (e.g., someone with crutches is brought a chair)?

4. How did the program transport SQ-LNS to the distribution site?

*Probes:*

- Are they stored onsite?

5. How is the distribution site arranged?

- Where are the participants waiting?
- What are participants doing as they wait?
- How is the crowd managed?

6. What is the distribution flow like?

- Are identification cards checked?
- Are the number of sachets counted before handing them to the caregiver?
- Is there a systematic way of entering and existing the collection/service delivery point?
- Do participants bring back empty LNS sachets?
- Where are the empty LNS sachets placed when turned in by participants?
- What happens to the empty LNS sachets at the end of the distribution day?

7. What services does the program provide in addition to SQ-LNS?

*[Information related to the SQ-LNS products, other maternal, infant, and young child nutrition information, anthropometric measurements, etc.]*

- What specifically do program staff tell program participants about SQ-LNS?
- Who provides these services? Please specify the role of each program staff.
- Are they provided at the time of distribution or at another time during the program life cycle?
- Do the services vary for children and pregnant and lactating women?

8. How does the program track information at the distribution site?

*[Number of sachets distributed, other services provided, such as anthropometric measurements, counseling, referral, etc.]*

- What tools are used?
- Are there different tools for children and pregnant and lactating women?
- What does the participant keep?
- What does the program keep?
- Who documents this information? Please note who writes on the participant card and who writes in the register.
- Does this vary for SQ-LNS and other services provided?
- If referrals (e.g., for children with severe or moderate acute malnutrition) are made, how are they tracked?

9. Did the program seek feedback on SQ-LNS from participants today?

*[Individually or in a group]*

- If yes, please describe how feedback was sought.

10. If you observed an interaction between program staff and participants, please note what participants said related to SQ-LNS or other services provided (or not provided). Please also note how the interaction went. For example, were the staff supportive, did they answer the participant’s questions, etc.?

11. In your opinion, could the program have done anything differently to improve the set-up, flow, communication related to SQ-LNS, etc. at this distribution site?

12. Time at distribution site

*Pick a child that arrives at the start, mid-point, and end of distribution. Note when the child and his/her caregiver entered and when they left the distribution site with the SQ-LNS ration. If the child/caregiver waits for others after they have received their SQ-LNS ration, then note that time as well as the time they physically left the site.*

| Time | Entry time  (time child/caregiver entered the distribution site) | Exit time  (time child/caregiver left the distribution site) | Notes |
| --- | --- | --- | --- |
| Start |  |  |  |
| Mid-point |  |  |  |
| End |  |  |  |

**Questions for partner staff**

13. How frequently do you distribute SQ-LNS at this site?

14. How does today compare with [the last distribution day (e.g., 4 weeks ago)]? With other sites?

*Probes:*

- Number of participants, flow, services provided, busier/quieter than usual, etc.
- Number of participants with a disability and accessibility for participants with disability.

15. What is working well with SQ-LNS distribution?

16. What could be improved with SQ-LNS distribution?

17. What is working well with the services (complementary activities) the program provides to participants along with SQ-LNS?

*Probes:*

- Information, anthropometric measurement, preventive health services

18. What do you think could be improved with the services the program provides to participants along with SQ-LNS?

*Probes:*

- Information and/or other SBC activities, anthropometric measurement, preventive health services
- For optimal distribution of SQ-LNS, what type(s) of support staff are required? What challenges are faced?

19. What feedback do participants give you related to SQ-LNS (product and how it is distributed)?

*Probes:*

- Positives and areas for improvement
- Is it possible to address them? Why or why not?
- Do they tell you anything about how SQ-LNS has affected them personally?

20. What feedback do participants give you related to other services (complementary activities) that the program provides at this distribution site?

*Probes:*

- Positives and areas for improvement
- Is it possible to address them? Why or why not?

21. If your organization were to expand the size of your SQ-LNS program, how many more children could you serve at this site?

*Probes:*

- What additional resources (staffing, space) would you need?
- What challenges would you face?

22. Do children or adults (including pregnant and lactating women) with disabilities regularly participate in the SQ-LNS program? Why or why not?

*Probes:*

- What (physical, attitudinal, informational, or policy) limits the participation of children and adults with a disability in SQ-LNS programs?
- What challenges have you experienced ensuring that children and caregivers with a disability are included in the SQ-LNS program?

23. Is there anything else you would like to share with us related to the distribution of SQ-LNS and other services the program provides?

**3. FGD guide (caregivers)**

Informed Consent

My name is ____ and I am working on behalf of USAID Advancing Nutrition. USAID Advancing Nutrition is a global nutrition project funded by the United States Agency for International Development (USAID). We are conducting a study in ___ to learn about USAID’s International Food Relief Partnership (IFRP)-funded programs that provide small quantity lipid-based nutrient supplement (SQ-LNS) programs to children 6–23 months of age. The results of this study will inform the design of future LNS programs.

During this group discussion, we would like to gather information about experiences and perspectives on the SQ-LNS program for children. The discussion will take about one hour and thirty minutes to complete. Your participation is entirely voluntary. You can decline to participate without losing any benefits that you receive from the SQ-LNS program, such as SQ-LNS or other related services. You are free to not answer certain questions or to stop participating at any time without any penalty. There is no direct benefit for participating.

We will keep any personal information that you give us, such as your name, confidential and will only share it in the study team. The study team does not include USAID. We will remove your personal information before sharing the information you give us outside the study team. In study reports and presentations, we will combine the information you provide us with the information we gather from others. Approximately ___ people will participate in this study. Please do not share the name and responses of other participants with anyone outside this group. We will share the combined information with USAID and may share it with government officials if they wish to see it.

Do you have any questions about participating?

- If yes, answer any questions.
- If no, move to the next question.

Do you agree to participate?

- If yes, thank them for agreeing to participate and move to the next question. For focus group discussions, ensure that each person agrees to participate.
- If no, thank them for their time and politely leave.

Can we audio record the conversation?

- If yes, proceed with audio recording. For focus group discussions, ensure that each person agrees.
- If no, say that it is no problem and proceed without audio recording.

If you have any questions about the study, you may contact [the study manager] at ____.

| ID (LNS_Country_FGD_Sequence#) |  |
| --- | --- |
| Location |  |
| Number of participants |  |
| Date of FGD |  |
| Facilitator name |  |
| Notetaker name |  |
| Language FGD conducted in |  |
| Consent to participate in FGD (Y/N) |  |
| Consent to audio record (Y/N) |  |

Note: Please assign a number to each participant and maintain the same number throughout the discussion.

**Questions**

1. Who eats the SQ-LNS product (mention local name)?

*Probes:*

- How old are the children who eat SQ-LNS?
- For how many months do the children eat SQ-LNS?

2. How do caregivers feed the SQ-LNS product to children?

*Probes:*

- As is or mixed with food?
- If mixed with milk, what type of milk (animal or formula)? If formula, what type of water do they use to prepare the formula?
- When do caregivers feed SQ-LNS to children (time of day, during meals, as a snack)?
- How frequently do caregivers feed SQ-LNS to children?
- Who else feeds SQ-LNS to children?
- Are there days caregivers do not prefer to feed SQ-LNS to children?
- Do children usually finish the whole sachet? Do they consume it all in one sitting or over the course of the day? Does this vary by age?
- What do caregivers do with the leftover SQ-LNS if the child does not finish it?
- What do caregivers do with the empty SQ-LNS sachets?

3. Why do caregivers give SQ-LNS to children?

*Probes:*

- What changes (e.g., length/height, weight, appetite, food preferences, less hungry, less cranky) in their children who eat SQ-LNS, if any, can the caregivers describe?
- What changes to them personally, if any, do caregivers (moms) describe?

4. What do caregivers like about SQ-LNS?

*Probes:*

- Taste, color, convenience, size, smell

5. What do children like about LSN-SQ?

*Probes:*

- Taste, color, convenience, size, smell

6. Is there anything children do not like about SQ-LNS?

*Probes:*

- Do some children have difficulty consuming their portion?
- How do caregivers usually handle these difficulties?
- Do children ever experience any side effects with the product, (e.g., vomiting, stomach upset)? Does this vary by age? How do caregivers handle this?

7. Is there anything that needs to be different about SQ-LNS?

*Probes:*

- Taste, color, packaging, size, smell

8. What do others (family, friends, and community) say about giving SQ-LNS to children?

*Probes:*

- Does anyone disapprove of giving SQ-LNS to children?

9. How often are SQ-LNS sachets distributed?

*Probes:*

- Where at home do caregivers store SQ-LNS?
- Which family members can access the SQ-LNS from where they are stored?
- How long does the number of SQ-LNS sachets received today last?

10. What do caregivers like about how SQ-LNS is distributed?

*Probes:*

- Frequency, site (common location vs. home delivery), duration, target age

11. What would caregivers like to be different about how SQ-LNS is distributed?

*Probes:*

- Frequency, site (common location vs. home delivery), duration, target age
- Advice or support given about how to use SQ-LNS
- Does this vary by season?
- Are there challenges with coming to the distribution site?
- Is saving sachets for the next distribution a burden?

12. What do caregivers like about the services that the program provides along with SQ-LNS?

*Probes:*

- Which services does the program provide?
- Anthropometric measurement (height, weight, MUAC), counseling, information related to SQ-LNS, information related to other maternal, infant, and young child nutrition, preventive health services
- What do caregivers like about these services?

13. How could the program improve the services provided with SQ-LNS?

*Probes:*

- Who provides the services?
- Where the program provides the services?
- Additional services?

14. Do children or adults with disabilities from your community regularly participate in the SQ-LNS program? Why or why not?

*Probes:*

- What limits the participation of children or caregivers who have a disability?
- What could be done to make the SQ-LNS program more accessible to adults or children with a disability in your community?

15. Is there anything else that you would like to share with us about the LNS product and how it is distributed?

**4. FGD guide (PLW)**

Informed Consent

My name is ____ and I am working on behalf of USAID Advancing Nutrition. USAID Advancing Nutrition is a global nutrition project funded by the United States Agency for International Development (USAID). We are conducting a study in ___ to learn about USAID’s International Food Relief Partnership (IFRP)-funded programs that provide small quantity lipid-based nutrient supplement (SQ-LNS) programs to children 6–23 months and pregnant and lactating women. The results of this study will inform the design of future LNS programs.

During this group discussion, we would like to gather information about experiences and perspectives on the SQ-LNS program for pregnant and lactating women. The discussion will take about one hour and thirty minutes to complete. Your participation is entirely voluntary. You can decline to participate without losing any benefits that you receive from the SQ-LNS program, such as SQ-LNS or other related services. You are free to not answer certain questions or stop participating at any time without any penalty. There is no direct benefit for participating.

We will keep any personal information that you give us, such as your name, confidential and will only share it in the study team. The study team does not include USAID. We will remove your personal information before sharing the information you give us outside the study team. In study reports and presentations, we will combine the information you provide us with the information we gather from others. Approximately ___ people will participate in this study. Please do not share the name and responses of other participants with anyone outside this group. We will share the combined information with USAID and may share it with government officials if they wish to see it.

Do you have any questions about participating?

- If yes, answer any questions.
- If no, move to the next question.

Do you agree to participate?

- If yes, thank them for agreeing to participate and move to the next question. For focus group discussions, ensure that each person agrees to participate.
- If no, thank them for their time and politely leave.
- Can we audio record the conversation?
- If yes, proceed with audio recording. For focus group discussions, ensure that each person agrees.
- If no, say that it is no problem and proceed without audio recording.

If you have any questions about the study, you may contact [the study manager] at ____.

| ID (LNS_Country_FGD PLW_Sequence#) |  |
| --- | --- |
| Location |  |
| Number of participants |  |
| Date of FGD |  |
| Facilitator name |  |
| Notetaker name |  |
| Language FGD conducted in |  |
| Consent to participate in FGD (Y/N) |  |
| Consent to audio record (Y/N) |  |

Note: Please assign a number to each participant and maintain the same number throughout the discussion.

**Questions**

1. Who eats the SQ-LNS product?

*Probes:*

- What stage of pregnancy/lactation (e.g., 5 months pregnant or 3 months post-delivery) do women begin eating SQ-LNS?
- How long do pregnant and lactating women eat the SQ-LNS product?

2. How do pregnant and lactating women eat the SQ-LNS product?

*Probes:*

- Do they mix the SQ-LNS with other food when they eat it?
- When do they eat SQ-LNS (time of day, mixed with meals, as a snack/in between meals)?
- How frequently do they eat SQ-LNS?
- Do they usually finish the whole sachet? Do they consume it all in one sitting or over the course of the day?
- What do they do with the empty SQ-LNS sachets?

3. Why do pregnant and lactating women eat SQ-LNS?

*Probes:*

- What changes (e.g., weight, appetite, food preferences), if any, do pregnant and lactating women describe?
- What differences, if any, do pregnant and lactating women describe in their newborn children after eating SQ-LNS products compared with a previous pregnancy when they did not eat SQ-LNS?
- What other benefits have pregnant and lactating women experienced from consuming SQ-LNS?

4. What do pregnant and lactating women like about SQ-LNS?

*Probes:*

- Taste, color, convenience, size, texture/consistency, smell

5. Is there anything pregnant and lactating women do not like about SQ-LNS?

*Probes:*

- Do they have any difficulties consuming their portion? How do they usually handle these difficulties?
- Have they ever experienced any side effects with the product, like vomiting, stomach upset?

6. Which special foods or supplements (e.g., iron and folic acid, fortified blended flour), in addition to SQ-LNS, do pregnant and lactating women eat?

*Probes:*

- Why do they eat these special foods or supplements in addition to SQ-LNS?

7. What do others (family, friends, and community) say about pregnant and lactating women eating SQ-LNS?

8. Is there anything pregnant and lactating women would like to be different about SQ-LNS?

*Probes:*

- Taste, color, packaging, size, texture/consistency, smell

9. How often do pregnant and lactating women come to get SQ-LNS sachets?

*Probes:*

- Where at home do they store SQ-LNS?
- Which family members access the SQ-LNS from where they have stored it?
- How long does the number of SQ-LNS sachets pregnant and lactating received today last?

10. What do pregnant and lactating women like about how SQ-LNS is distributed?

*Probes:*

- Frequency, site (common location vs. home delivery), duration, target stage of pregnancy

11. What would pregnant and lactating women like to be different about how SQ-LNS is distributed?

*Probes:*

- Frequency, site (common location vs. home delivery), duration, target age
- Advice or support given about how to use SQ-LNS? Or what to do if they have difficulties eating it?
- Are there challenges with coming to the distribution site?
- Does the distribution need to vary by season (e.g., rainy season)?
- Is saving sachets for the next distribution a burden?

12. Which services does the program provide along with the SQ-LNS?

*Probes:*

- Anthropometric measurements (weight, height, MUAC), counseling, information related to SQ-LNS, information related to other maternal, infant, and young child nutrition, preventive health services
- What do pregnant and lactating women like about the services the program provides along with SQ-LNS?
- What benefits have pregnant and lactating women experienced from participating in this program (SQ-LNS with other services)?

13. How could the program improve the services provided with SQ-LNS?

*Probes:*

- Who (organization, gender, etc.) provides the services?
- Where the program provides the services? Length of time?
- Additional services?

14. Do pregnant and lactating women with disabilities from your community regularly participate in the SQ-LNS program? Why or why not?

*Probes:*

- What limits participation of pregnant and lactating women with a disability?
- What could be done to make the SQ-LNS program more accessible to adults or children with a disability in your community?

15. Is there anything else you would like to share with us about the LNS product and how it is distributed?

**5. Sub-themes for Data Analysis**

The sub-themes for implementation were geographic scope; coordination; delivery platform; human resources; enrollment; duration of supplementation; frequency of distribution; complementary activities; social and behavior change; monitoring and evaluation; logistics successes; challenges; and recommendations. The sub-themes for product use were consumption practices; benefits; and recommendations. The sub-themes for scale-up were scope and considerations for scale-up.

1. Defined as government, United Nations agencies, nongovernmental organizations (national or international), institutions (academic or non-academic), or private sector working in the field of nutrition. [↑](#footnote-ref-1)
2. Defined as goods/inputs or services provided with SQ-LNS to improve the overall well-being of children. [↑](#footnote-ref-2)
